# Task load modulates network interactions between bilateral fronto-parietal and cerebellar areas during verbal working memory

**DOI:** 10.1016/j.isci.2026.115502

**Published:** 2026-03-26

**Authors:** Sabrina Turker, Gerasimos Gerardos, Felix Büch, Beatrice Fumagalli, Philipp Kuhnke, Gesa Hartwigsen

**Affiliations:** 1 Cognitive and Biological Psychology, Wilhelm Wundt Institute for Psychology, Leipzig University, Leipzig, Germany; 2 Research Group Cognition and Plasticity, Max Planck Institute for Human Cognitive and Brain Sciences Leipzig, Leipzig, Germany; 3 Brain and Language Lab, Department for Behavioral and Cognitive Biology, University of Vienna, Vienna, Austria; 4 Clinical Psychology and Psychotherapy, Wilhelm Wundt Institute for Psychology, Leipzig University, Leipzig, Germany; 5 Interdisciplinary Graduate Program in the Brain and Mind Sciences, University of Crete, Heraklion, Crete, Greece

**Keywords:** Neuroscience, Cognitive neuroscience

## Abstract

Verbal working memory (VWM) relies on frontal and parietal regions, yet their functional interactions, particularly with the cerebellum, remain underexplored. Prior studies link higher VWM load to increased parieto-prefrontal coupling and associate stronger interactions with better performance. This suggests that connectivity may underlie individual differences in efficiency. However, fronto-parieto-cerebellar dynamics have not been systematically examined to date. We investigated activation and directed functional coupling between six pre-selected regions of interest during a VWM task. Higher task load increased activity in fronto-parietal regions and the cerebellum. Connectivity analyses revealed bidirectional facilitatory interactions between fronto-parietal regions, but reduced cerebello-cortical coupling as demands increased. Positive left parieto-frontal coupling with increasing load showed links to performance, indicating that less interaction was required for good performance. These effects were consistent across two sessions. Our study highlights that fronto-parietal coupling adapts to support increasing task demands, whereas reduced cerebello-cortical interactions suggest a distinct role for the cerebellum.

## Highlights

• Fronto-parietal and cerebellar activity increase with higher working memory load
• Higher working memory demands increase bi-directional fronto-parietal facilitation
• Higher working memory demands reduce cerebello-cortical coupling
• Activation and connectivity effects remain stable across two sessions

## Graphical abstract

**Figure:**
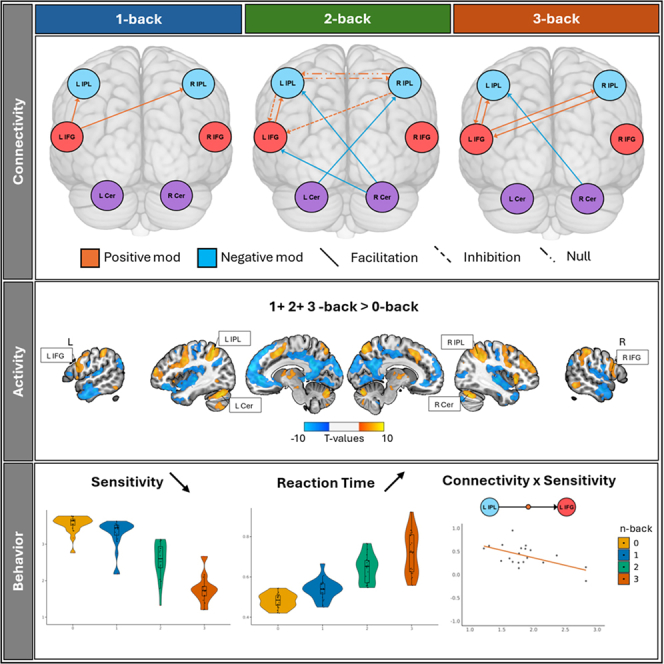

## Introduction

A key capacity of human cognition is the ability to store, maintain, and manipulate information under ever-changing conditions, which is enabled by working memory. Working memory is crucial for goal-directed behavior, plays a central role in daily life activities, and is predictive for a wide range of higher-level cognitive measures, including problem-solving, language acquisition, and reading comprehension.^1^^,^^2^^,^^3^ Working memory functions are known to decline with age^4^^,^^5^^,^^6^ and are impaired in several neurological and psychiatric disorders.^7^^,^^8^ Such impairments can severely disrupt daily activities and communication, highlighting the critical role of working memory for everyday functioning.

Cognitive models assume that working memory entails separate components, including domain-specific mechanisms of memory maintenance and domain-general mechanisms of executive control.^9^^,^^10^ Verbal working memory (VWM), the temporary maintenance of verbal information, is particularly relevant for successful and efficient everyday communication.^11^ Despite decades of research, however, the exact processing mechanisms, especially on the neural level, remain poorly understood.^12^^,^^13^

Based on lesion and neuroimaging studies, modern views argue that VWM largely overlaps with the language network.^14^ Specifically, meta-analytic studies have demonstrated that the underlying neural correlates of VWM engage a large network of cortical and subcortical regions, with a key role of prefrontal and parietal structures, and a strong contribution of cerebellar and basal ganglia regions.^15^^,^^16^^,^^17^^,^^18^ Based on these studies, the left inferior parietal lobe (IPL) has been associated with phonological storage processes,^19^^,^^20^^,^^21^ while the left inferior frontal gyrus (IFG) likely represents a key region for phonological rehearsal.^21^^,^^22^

Aside from fronto-parietal regions, the cerebellum is also believed to play a crucial role in VWM.^23^ Rather than serving only domain-general control, cerebellar involvement in VWM has been linked to internal-forward models^24^ and error-based prediction functions: For example, the cerebellum predicts upcoming phonological items during VWM tasks^25^ and is sensitive to linguistic prediction and error-monitoring in language comprehension.^26^^,^^27^ Classic VWM accounts propose that during subvocal rehearsal, the cerebellum generates predictive models of articulatory commands and compares them with sensory consequences to detect and correct mismatches in phonological traces.^25^^,^^28^^,^^29^ Crus I and II have been consistently implicated in higher-order linguistic and emotional processing as well as phonological manipulation^15^^,^^30^^,^^31^ while lobule VI has been shown to scale its activity with VWM load, providing additional support for error correction and temporal prediction during rehearsal processes.^23^^,^^32^

Whereas regional cerebellar activation in VWM is well-documented, far less is known about how these cerebellar nodes interact directionally with cortical regions as task demands increase. One recent study explored the interaction between parietal and frontal areas during VWM tasks, demonstrating increased interaction between the right parietal cortex and the ipsilateral dorsolateral prefrontal cortex during increasing task demands.^33^ Moreover, stronger parieto-prefrontal interactions with increasing task load were associated with more efficient task performance in this study. These findings suggest that individual differences in coupling strength between core VWM nodes could predict differences in cognitive abilities and thereby affect VWM task performance. Yet, how these regions interact with the cerebellum remains to be explored.

Although previous studies support a strong engagement of parieto-prefrontal areas in VWM, it is less clear how variations in task load modulate interactions within the larger VWM network, including parietal, prefrontal, and cerebellar regions. The present study was designed to address this gap. In the present work, we tested the hypothesis that cerebellar subregions contribute to VWM by generating predictive models and adjusting cortical representations via feedback signals. Moreover, we asked how directed coupling between core VWM nodes, including the cerebellum, responds to increasing VWM load and how the direction of task-related modulations (i.e., facilitation or inhibition) may be linked to behavioral performance. Additionally, we tested the reliability of these effects by elucidating session effects on the behavioral and neural levels. To foreshadow our main results, we found that, as expected, higher task load was linked to higher task-related activity in distributed task-positive areas, which were found to strongly overlap with the multiple demand network (MDN). They include bilateral fronto-parietal regions and the cerebellum. With respect to task-related network interactions, increasing VWM load was mainly characterized by increasing bidirectional, facilitatory interactions between fronto-parietal areas and decreased cerebello-cortical connectivity. Increased positive coupling between the left inferior parietal and frontal cortex with increasing task load was directly linked to performance, with a negative correlation indicating that less modulation was required for good performance. These effects were overall stable across sessions. Collectively, these findings emphasize the relevance of distributed interactions between parietal, frontal, and cerebellar areas, with a key role of left parieto-frontal interactions.

## Results

### Behavioral performance

Participants showed significant differences in sensitivity and response times for the different task conditions, as revealed by a Friedman rank-sum test (χ^2^ = 57.84, *p* < 0.001). Sensitivity was higher for the 0-back (control condition; *M* = 3.6 ± 0.2) than for the 1-back (*M* = 3.37 ± 0.4), 2-back (*M* = 2.71 ± 0.53), and the 3-back task (*M* = 1.86 ± 0.42). Post-hoc Wilcoxon signed-rank tests confirmed significant differences in sensitivity between all conditions (0-back vs. 1-back: *p* = 0.005, all other comparisons *p* < 0.001 after Bonferroni correction). An aligned rank transform ANOVA confirmed a significant main effect of n-back condition (*F*_(3,133)_ = 221.64, *p* < 0.001), but no significant effect of session (*F*_(1,133)_ = 1.79, *p* = 0.183) and no significant n-back condition by session interaction (*F*_(3,133)_ = 0.61, *p* = 0.607) for sensitivity scores.

**Figure 1:**
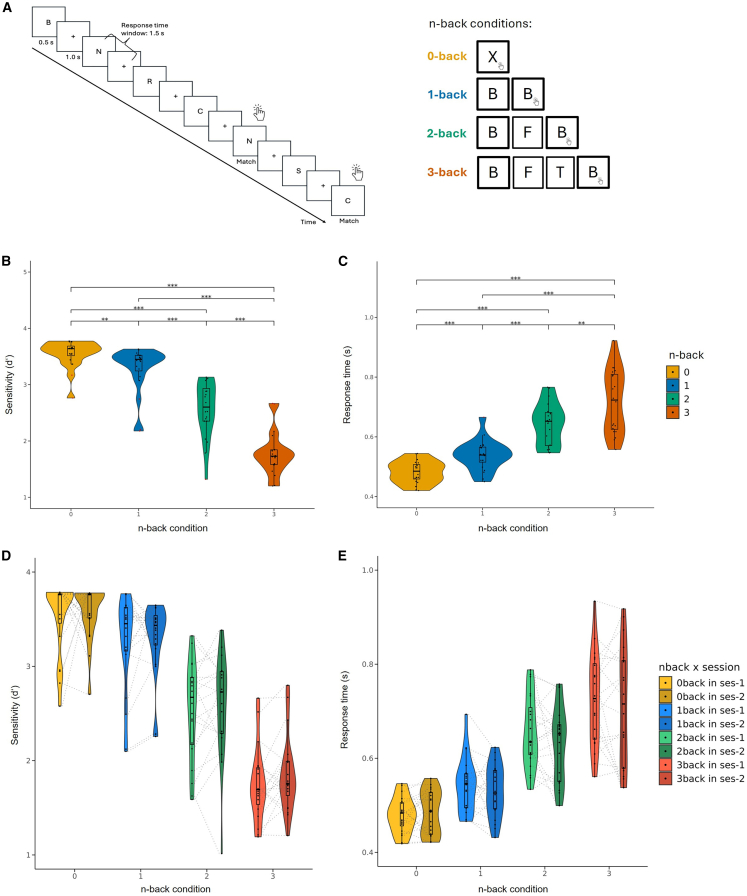
Experimental design and behavioral results (A) Visualization of the n-back task and the included n-back conditions. (B) Mean sensitivity *d’* per n-back condition across participants. (C) Mean response times in seconds per n-back task across participants. (D) Stability of performance in terms of sensitivity (d') across two sessions. (E) Stability of performance in terms of response time in seconds across two sessions. Results for both sensitivity and response time show significant differences across n-back conditions, with the 3-back condition having the lowest sensitivity and the longest response times. No significant differences in performance between sessions were found. Violin plots in (B) to (E) show the distribution of the data with embedded boxplots indicating the median and interquartile range (IQR). Whiskers represent 1.5×IQR and points represent individual observations. Moreover, lines connect repeated measurements from the same participant across sessions in (D) and (E). Significance levels are provided with asterisks (∗∗∗*p* < 0.001 and ∗∗*p* < 0.01), and outliers are shown in red.

Similarly, participants’ response times were significantly different across conditions (Friedman rank-sum test: χ^2^ = 52.5, *p* < 0.001). Response times were faster during the 0-back (*M* = 0.48 ± 0.01) than the 1-back (*M* = 0.54 ± 0.05), 2-back (*M* = 0.64 ± 0.01), and 3-back task (*M* = 0.72 ± 0.11). Post-hoc Wilcoxon signed-rank t-tests confirmed significant differences in response times between the respective conditions (2-back vs. 3-back: *p* = 0.007, all other comparisons *p* < 0.001 after Bonferroni correction). An aligned rank transform ANOVA confirmed a significant main effect of condition (*F*_(3,133)_ = 180.00, *p* < 0.001), but no significant effect of session (*F*_(1,133)_ = 1.00, *p* = 0.32) and no significant n-back condition by session interaction (*F*_(3,133)_ = 0.53, *p* = 0.663) (see also Figures 1B–1E). The ANOVA results are summarized in Table S1. Regarding session effects, neither sensitivity nor response times showed significant effects for any condition (all *p* > 0.05), i.e., participants did not get better or faster the second time they did the task.

### Functional activation

**Figure 2:**
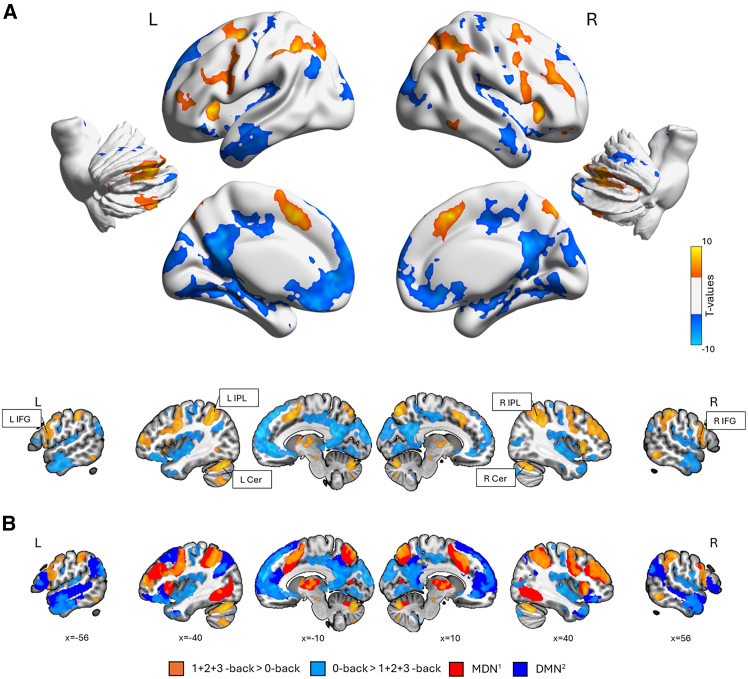
Task-related activity during VWM (A) Brain areas that show higher activity for VWM tasks (1+2+3-back) vs. the control task (0-back). Higher activation for the VWM tasks covers classical VWM regions like the bilateral IPL, portions of the bilateral IFG, the bilateral cerebellum and the prefrontal cortices. Results are shown at *p* < 0.001 uncorrected at the voxel level and *p* < 0.05 FWE-corrected at the cluster level. Selected regions are labeled. (B) Overlap of activation patterns with the MDN and the default mode network (DMN). MDN and DMN masks were taken from Darda et al. (2018)^34^ and Schaefer et al. (2018).^35^ L/R IFG = left and right inferior frontal gyrus, L/R IPL = left and right inferior parietal cortex, L/R CER = left and right cerebellum.

The three n-back conditions elicited higher activity in classical VWM areas, including the bilateral IPL, the bilateral IFG (specifically, portions of the posterior IFG), the bilateral precentral gyri, and the bilateral cerebellum (Figure 2A; Table 1). These activation patterns show meaningful overlap with the so-called (fronto-parietal) MDN, which is known to be crucial for coordinated, goal-directed behavior and sensitive to hierarchical task structure, relevant for most complex cognitive tasks.^36^ The overlap coefficient of the MDN and the positive task activation was 0.559, indicating that 55.9% of voxels of the task activation (19,940 voxels) overlapped with the MDN (28,761 voxels).

**Table 1:** Functional activation clusters showing higher activity for the VWM tasks (1 + 2+3-back > 0-back)

Cluster	Cluster size	HP	Brain area	MNI peaks			t -value
				x	y	z	
1	5029	L/R	frontal cortex and subcortex	4	20	48	11.1
				−30	2	62	10.7
				−30	26	−4	10.6
				−32	−2	62	10.6
				−26	−2	54	10.2
2	3853	R	frontal cortex	28	4	62	10.6
				32	26	−2	10.1
				38	20	0	10
				34	22	−4	9.79
				44	44	22	7.92
3	3420	R	inferior and superior parietal cortex	30	−66	36	14.2
				50	−34	56	12.8
				34	−48	44	11
				58	−30	50	9.53
				34	−52	42	9.5
4	3234	L/R	cerebellum (crus I, II, lobules VI, VIIb)	−28	−76	−52	11.3
				24	−64	−28	10.5
				−8	−78	−26	10.3
				−26	−64	−30	10.3
				28	−72	−52	10.1
5	3041	L	inferior and superior parietal cortex	−48	−46	48	13.2
				−44	−54	52	12.2
				−46	−50	42	11.6
				−36	−52	50	11.5
				−28	−48	38	9.58
6	446	L	frontal pole	−40	56	12	8.62
				−40	54	18	7.04
				−44	42	4	5.72
7	260	R	temporo-occipital cortex	54	−56	−12	7.82
8	211	L	temporo-occipital cortex	−54	−56	−16	4.72
9	210	R	thalamus	6	−20	−2	6.69
				8	−8	2	6.65
				0	−26	0	6.16
10	159	R	basal ganglia (putamen, caudate, pallidum)	22	4	4	7.85
				16	4	14	5.55
				14	12	6	5.47
				18	12	16	5.3
				12	8	2	4.72
11	77	R	frontal pole	22	44	−16	5.24
				30	50	−16	5.22
				26	48	−10	4.43

For each cluster, a maximum of 5 peak coordinates is presented. Overall, activation is spread bilaterally, with the strongest contributions of the frontal cortices and the parietal cortices, as well as the bilateral cerebellum. Highest activation for the respective n-back conditions was achieved in a cluster covering large portions of the left frontal cortex and portions of subcortical regions (e.g., thalamus). Coordinates are provided in MNI space, cluster sizes in voxels. HP = hemisphere (left/right).

During the 0-back task (0-back >1+2+3-back task), in contrast, higher activation was found in regions overlapping with the default mode network (DMN). The overlap coefficient of the DMN and the negative task activation was 0.372, showing that 37.2% of the voxels of the DMN (31,443 voxels) overlapped with the negative task activation (34,811 voxels). The DMN is known to decrease its activity during attention-demanding tasks and is considered a network reflecting the brain’s intrinsic activity.^37^^,^^38^ The DMN activity observed in this study comprised the bilateral temporal lobes, bilateral occipital cortices, the left superior frontal gyrus, and a large range of areas in medial brain areas in both hemispheres (see Figure 2B), in line with earlier research.^37^

**Figure 3:**
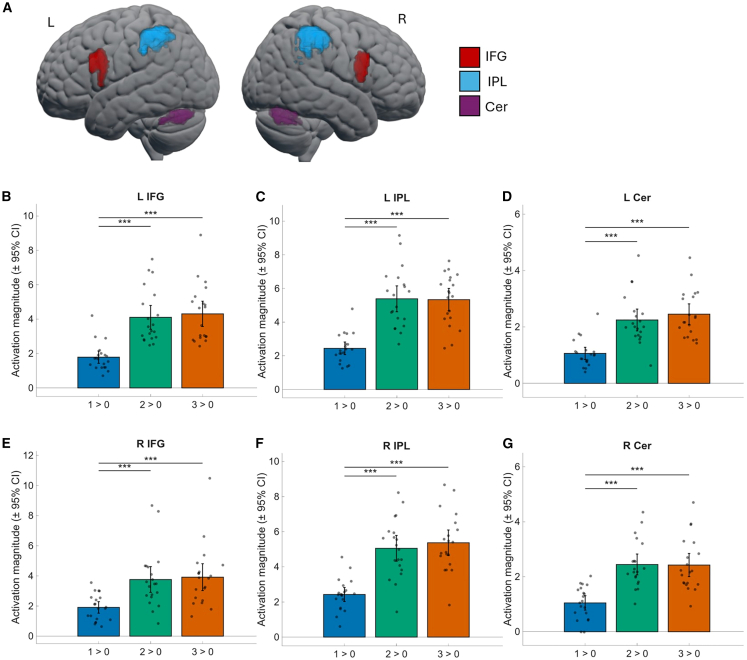
Activation magnitude in selected ROIs within the core VWM network (A) Selection of ROIs to investigate task load activation differences. (B–D) Left-hemispheric ROIs and their contributions to VWM. (E–G) Right-hemispheric ROIs and their contributions to VWM. Overall, the 1-back task elicited lower activation in all selected ROIs as compared to the 2- and 3-back tasks, respectively. However, no significant activation differences between the 2- and 3-back tasks were found. Bars represent mean activation magnitudes across participants, with error bars indicating 95% confidence intervals. Black dots represent individual participants. L/R IFG = left and right inferior frontal gyrus, L/R IPL = left and right inferior parietal cortex, L/R CER = left and right cerebellum. ∗∗∗*p* < 0.001.

We also explored differential activation patterns depending on the specific n-back conditions in six core VWM regions, namely the bilateral IFG, bilateral IPL and bilateral cerebellum. These were defined based on anatomical priors and constrained by data-driven information (see STAR Methods section for details and Figure S1 for an illustration). Activation magnitude in these six regions differed significantly depending on task difficulty (Figure 3; see also Figure S2 for an illustration of the parametric modulation of task load). The 1-back task elicited the lowest activation in all six regions, with comparable activation magnitude in the respective homologues (left and right IFG, left and right IPL, left and right cerebellum). The activation during the 1-back differed significantly from the 2-back and 3-back across all regions, but there were no significant differences in activation for the 2- and 3-back tasks, despite significant performance differences (see Figure 1).

### Task-based effective connectivity

The six core VWM regions (bilateral IPL, bilateral IFG, and bilateral cerebellum) were then selected as nodes to explore directed functional coupling during VWM. To simplify the presentation of the results, we first provide a brief overview of the results, followed by a presentation of modulations for the three separate n-back conditions and a summary of posterior-to-anterior and anterior-to-posterior connectivity modulations by task. Finally, direct links between task-related connectivity and behavior are presented.

**Figure 4:**
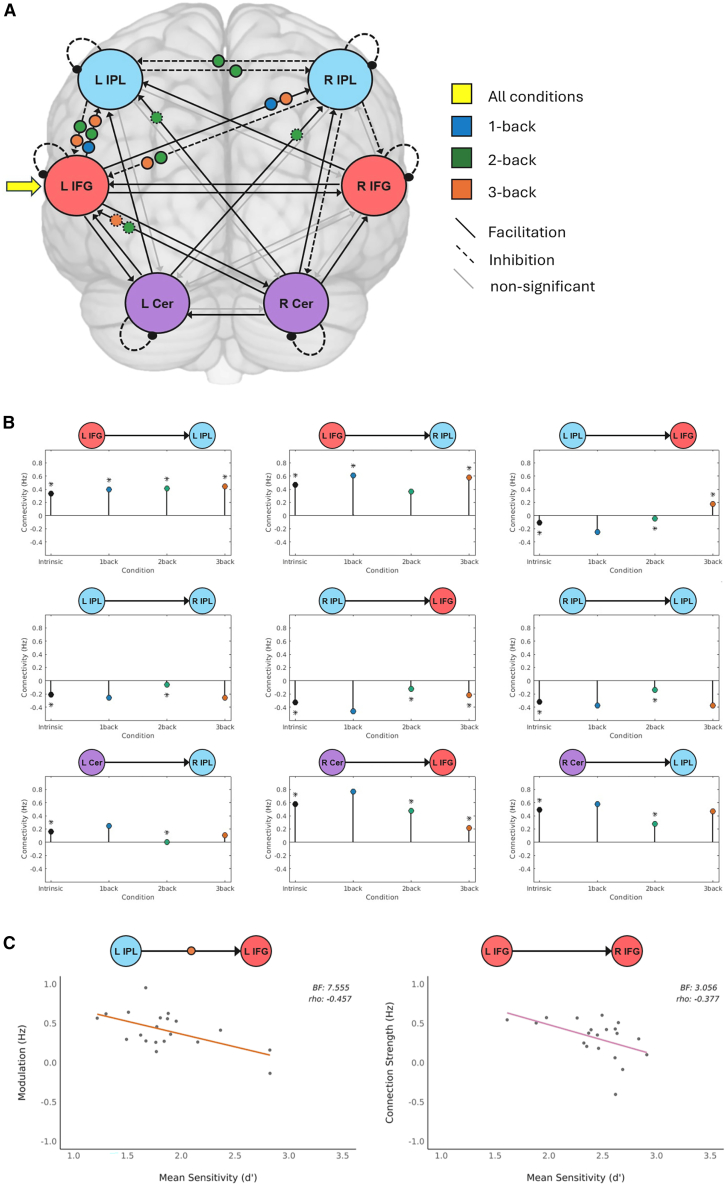
Effective connectivity during the VWM task (A) Significant modulations of connections by task condition. Intrinsic connectivity between the respective regions is either positive (facilitatory; solid line) or negative (inhibitory; dotted line). Additionally, task effects on intrinsic connections are displayed: facilitation (positive effect of task) and inhibition (negative effect of task) as solid lines or dotted lines around the respective modulation. (B) Detailed visual representation of intrinsic and final connectivity after modulation for all tasks. Significant modulations (visualized in A) are highlighted (∗). Connectivity values are provided in Hz. (C) Moderate Bayesian correlations (BF_10_ > 3) between effective connectivity and task performance (LIPL to LIFG 3-back modulation with mean sensitivity *d’* in the 3-back task; LIFG to RIFG intrinsic connectivity with mean sensitivity *d’* across all n-back tasks).

Overall, this pre-defined VWM network showed robust intrinsic functional coupling during the different n-back tasks. Notably, whereas all other intrinsic connections between the regions were facilitatory, both IPLs consistently exerted inhibitory influences on the rest of the network. In addition, the left IFG was the only region to receive the driving task input, underscoring its pivotal role in VWM. However, connections were differently modulated by specific n-back conditions, and these were linked to behavior (see Figure 4; Table 2; for an illustration of the full DCM model, please see Figure S3A).

**Table 2:** Results of the effective connectivity analysis

	Intrinsic Conn.	1-back		2-back		3-back	
		Mod.	Conn.	Mod.	Conn.	Mod.	Conn.
L IFG -> L IFG	**−0.806**	0	0	0	0	0	0
R IFG -> R IFG	**−0.396**	0	0	0	0	0	0
L IPL -> L IPL	**−0.087**	0	0	0	0	0	0
R IPL -> R IPL	**−0.119**	0	0	0	0	0	0
L CER -> L CER	**−0.555**	0	0	0	0	0	0
R CER -> R CER	**−0.965**	0	0	0	0	0	0
L IFG - > R IFG	**0.301**	0	0.301	0	0.301	0	0.301
L IFG -> L IPL	**0.332**	**0.239**	**0.401**	**0.250**	**0.411**	**0.280**	**0.441**
L IFG -> R IPL	**0.466**	**0.244**	**0.609**	0	0.365	**0.214**	**0.579**
L IFG -> L CER	**0.111**	0	0.111	0	0.111	0	0.111
L IFG -> R CER	**0.302**	0	0.302	0	0.302	0	0.302
R IFG -> L IFG	**0.182**	0	0.182	0	0.182	0	0.182
R IFG -> L IPL	**0.185**	0	0.185	0	0.185	0	0.185
L IPL -> L IFG	**−0.109**	0	−0.249	**0.204**	**−0.045**	**0.427**	**0.178**
L IPL -> R IPL	**−0.211**	0	−0.255	**0.194**	**−0.060**	0	−0.255
R IPL -> L IFG	**−0.331**	0	−0.458	**0.336**	**−0.123**	**0.238**	**−0.220**
R IPL -> L IPL	**−0.321**	0	−0.373	**0.233**	**−0.140**	0	−0.373
R IPL -> R CER	**−0.170**	0	−0.17	0	−0.17	0	−0.17
L CER -> L IFG	**0.150**	0	0.15	0	0.15	0	0.15
L CER -> L IPL	**0.113**	0	0.113	0	0.113	0	0.113
L CER -> R IPL	**0.163**	0	0.251	**−0.248**	**0.003**	−0.147	0.104
R CER -> L IFG	**0.578**	0	0.764	**−0.287**	**0.477**	**−0.550**	**0.214**
R CER -> R IFG	**0.221**	0	0.221	0	0.221	0	0.221
R CER -> L IPL	**0.489**	0	0.581	**−0.304**	**0.277**	−0.111	0.470
R CER -> R IPL	**0.308**	0	0.308	0	0.308	0	0.308
R CER -> L CER	**0.176**	0	0.176	0	0.176	0	0.176

We report significant intrinsic connections and significant modulations of connections between the ROIs by the n-back condition. For each significant modulation (Mod., task-specific modulation relative to the intrinsic connectivity value), the resulting connectivity value (Conn., reflecting the intrinsic connectivity and task modulation) is provided in Hz. Please note that only significant findings with a posterior probability of > 0.99 are presented. All significant effects are highlighted in **bold**. L/R IFG = left and right inferior frontal gyrus, L/R IPL = left and right inferior parietal cortex, L/R CER = left and right cerebellum.

#### Connectivity modulations by task condition

For the 1-back task with the lowest VWM demands, only increased fronto-parietal interactions were observed (L IFG → L/R IPL). For the 2-back task, we observed (1) increased left fronto-parietal facilitation (L IFG → L IPL), (2) decreased parieto-frontal inhibition (L IPL → L IFG, R IPL → L IFG), (3) decreased cerebello-cortical connectivity (R Cer → L IFG, R Cer → L IPL, L Cer → R IPL), and (4) a neutralization of inhibition between the IPLs (L IPL → R IPL, R IPL → L IPL). For the 3-back task, all fronto-parietal connections showed a significant increase in connectivity (L IFG → L IPL, L IFG → R IPL), and the intrinsic inhibition of the bilateral IPLs on the left IFG was turned into facilitation (L IPL → L IFG, R IPL → L IFG).

#### Task-dependent changes in coupling between anterior and posterior regions

Apart from looking at modulations by task, it is important to consider changes in posterior-to-anterior and anterior-to-posterior communication between core VWM nodes. Regarding posterior-to-anterior communication, the bilateral cerebellum had an intrinsic, facilitatory influence on other VWM nodes, which was decreased during the more challenging 2- and 3-back conditions. Parieto-frontal coupling showed task load effects as well, with the intrinsic inhibition of the left IPL on the left IFG being decreased during the 2-back task and turning into facilitation during the 3-back task. Significant cross-hemispheric posterior-to-anterior modulations were found between the right IPL and the left IFG. The intrinsic inhibitory influence from the right IPL on the left IFG was reduced during the 2-back and turned into facilitation during the 3-back task.

Further investigating anterior-to-posterior communication, we found that anterior-to-posterior functional coupling was only modulated by task in the case of the influence of the left IFG on the bilateral IPL. Effective connectivity from the left IFG to the left IPL showed a parametric modulation: the more difficult the task, the larger the facilitatory coupling. In the case of cross-hemispheric anterior-to-posterior coupling, the left IFG exerted a facilitatory influence on the right IPL, which increased during the 1- and 3-back tasks.

#### Connectivity-behavior relationships

While increasing task demands in the 3-back condition turned the inhibitory intrinsic coupling from left IPL to left IFG into facilitation, this increase was negatively linked with performance. Accordingly, brain-behavior correlations revealed that a stronger positive modulation of left IPL to left IFG coupling during the 3-back task was related to decreased performance (BF_10_ = 7.555, *rho* = −0.457). In other words, less positive coupling was linked to better behavioral sensitivity, likely reflecting higher efficiency (Figure 4C). Likewise, stronger positive intrinsic coupling from left to right IFG was associated with overall decreased performance (BF_10_ = 3.056, *rho* = −0.377), reflecting reduced behavioral efficiency. To control for between-participant differences in overall task performance, we mean-centered sensitivity (d′) values within each participant by subtracting each participant’s average sensitivity (d') across all conditions, weighted by the frequency of each condition. This procedure removes variance associated with general ability while preserving within-subject fluctuations across task conditions. After mean-centering, the negative correlation between IPL → IFG effective connectivity and behavioral performance was no longer credible (BF10 = 1.026, weak), indicating that the relationship was primarily driven by between-subject differences in overall task performance rather than condition-specific fluctuations.

**Figure 5:**
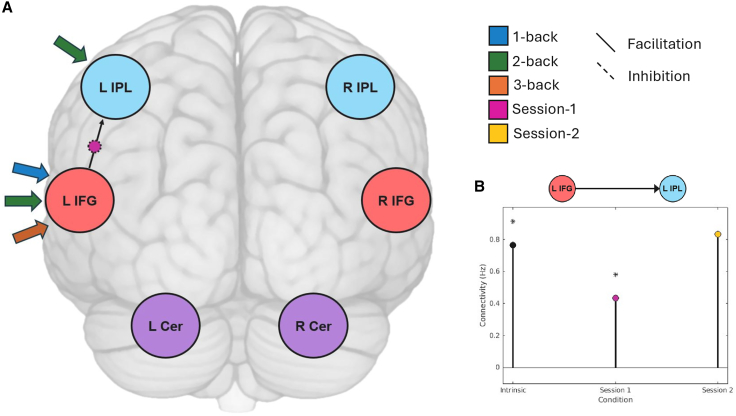
Session effects on effective connectivity (A) Results of the DCM explore the effect of session on effective connectivity within the VWM network. Only connectivity from the left IFG to the left IPL showed significant session effects, with a significant decrease of this facilitatory connection during session 1, which was neutralized in session 2. (B) Direct comparison of connectivity (in Hz) for sessions 1 and 2. Significant effects are highlighted (∗).

To investigate possible session effects on the network level, we checked modulations by session on the investigated DCM network (Figure 5; for an illustration of the full DCM model, please see Figure S3B). We found that only connectivity from the left IFG to the left IPL was modulated by task session, with intrinsic facilitation decreasing significantly during the first n-back task session and increasing again during the second n-back session. To put it differently, doing the n-back task for the first time led to a significant decrease in left IFG to left IPL coupling. When participants did the task for the second time, the connectivity was not different from the intrinsic value but showed a significant difference from the first session. The driving input for all 1-, 2-, and 3-back conditions originated in the left IFG, underscoring its pivotal role in VWM. Additionally, the left IPL served as a driving input specifically for the 2-back condition, supporting the role of the IPL, alongside the IFG, as part of the frontoparietal network supporting VWM.

## Discussion

**Figure 6:**
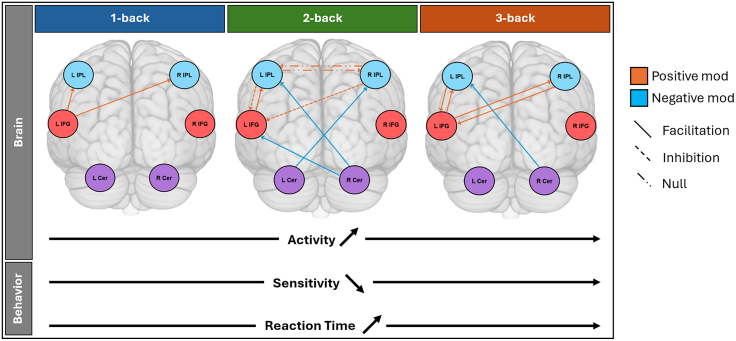
Summary of the main findings Increasing task load led to significant changes in task-related connectivity, activity, and behavior. Effective connectivity was characterized by changes in positive and negative modulations, reflecting facilitation and inhibition between core areas for VWM (mod = modulation).

In the present work, we investigated the neural mechanisms underlying VWM through a combination of behavioral, functional brain activation, and effective connectivity measures. We focused on contributions of and interactions between frontal, parietal, and cerebellar regions for increasing VWM load. We also assessed variability versus stability of performance and activity across sessions. The four key findings of this study are as follows (summarized in Figure 6):

First, we found stable VWM performance across two sessions, with increasing task load consistently leading to lower sensitivity and higher reaction times, with no evidence for significant learning effects for the performed n-back tasks.

Second, task-related activation for the n-back tasks as compared to a baseline (0-back) showed large overlap with the task-positive MDN, including activation in the bilateral IFG, bilateral IPL, and the bilateral cerebellum. The opposite contrast, comparing the baseline with the n-back tasks, engaged distributed areas overlapping with the task-negative DMN.

Third, the VWM network depends crucially upon the interaction between the left IFG and the left IPL, with connectivity between these two areas showing robust task-load effects while being reversely linked to performance under higher task load, as reflected in decreased sensitivity with increased positive coupling. This suggests that while higher cognitive load requires facilitatory parieto-frontal interactions, higher behavioral sensitivity is linked to lower coupling, reflecting more efficient performance. The cerebellum, in contrast, showed inhibitory cross-hemispheric influences on frontal and parietal regions, demonstrating that increasing cognitive load requires a balance of inhibitory and facilitatory interactions at the larger network level.

Fourth, in the absence of performance changes between the first and second sessions, we observed an initial decrease in the positive coupling from left IFG to left IPL relative to the intrinsic connectivity pattern in the first session that normalized in the second session. These changes may reflect an initial inhibitory top-down strategy to solve the task that became less relevant upon experience with repetition.

Behavioral analyses of sensitivity, a combined measure of response times and accuracy, revealed consistent performance across sessions, indicating that task familiarity or repetition did not significantly influence behavioral outcomes over time. This is particularly relevant for neurostimulation studies exploring the impact of different protocols (e.g., effective versus sham stimulation) across different sessions or pre-versus post-stimulation effects. Participants performed close to ceiling on the control condition (0-back), indicating vigilance and engagement with the task. In contrast, when working memory load was increased (1-, 2-, and 3-back), notable within-task differences were observed based on cognitive load. As expected, response times increased, and sensitivity decreased with task difficulty. Specifically, participants performed best on the 1-back task and exhibited the poorest performance on the 3-back task, both in terms of response times and accuracy. The progressive decline in sensitivity across increasing VWM demands is consistent with the cognitive load hypothesis, demonstrating the limited capacity of the working memory system under higher demands.^39^^,^^40^^,^^41^

Univariate fMRI analyses revealed task-specific patterns of activation that broadly overlapped with well-characterized large-scale brain networks, notably the extended task-positive MDN,^42^ including the cerebellum, and the task-negative DMN (e.g., see summary in Menon, 2023^43^). MDN activation was observed primarily during higher cognitive load conditions (2-back and 3-back), supporting its role in goal-directed behavior, executive control, and VWM performance.^44^^,^^45^ This pattern is in line with previous studies describing the MDN as being up-regulated in response to increased task difficulty.^46^^,^^47^ Regions associated with the DMN are typically affected by the allocation of attentional resources to the task and are thus deactivated or suppressed during externally focused tasks.^43^ Here, they showed strong overlap with the control task, the 0-back task, which was less challenging than the 1- to 3-back tasks and required very little VWM. Overall, this is in line with previous findings, suggesting that during a VWM task, both the VWM network and the DMN are activated, but during execution, the DMN is suppressed.^48^ Taken together, our study corroborates earlier findings suggesting a dynamic interplay between task-positive and task-negative networks during VWM tasks, most likely reflecting cognitive reallocation or reconfiguration mechanisms necessary to manage increasing task demands. Although the cerebellum is not part of the “classic” MDN,^49^ more recent studies have associated cerebellar activity with increased task demands.^50^ The observed increases in task-related activity with increasing task load in the cerebellar subregions crus I/II in our study support the role of these areas for domain-general control functions,^51^ arguing for a strong association with the extended MDN. Strikingly, these areas exhibited inhibitory cross-hemispheric coupling on frontal and parietal regions during the more challenging task conditions, likely reflecting increased cognitive control demands.^52^

Examining individual activation strength in core VWM nodes revealed that the 1-back task elicited the lowest activation in all core VWM regions as compared to the 2- and 3-back tasks. Surprisingly, the 2- and 3-back tasks did not differ significantly in terms of activation, despite significant differences in behavioral performance, both in sensitivity and response times. This could speak in favor of a ceiling effect in the 2-back task, which had high task demands and thus already elicited the maximal activation magnitude. The 3-back task was highly challenging for most participants and yielded low accuracy and slow response times. The relevance of the contribution of the core VWM areas in the bilateral IFG, IPL, and cerebellum was further supported by the parametric modulation of their contribution with increasing task load.

In terms of directed functional coupling between VWM nodes, one of the most striking findings was that bi-directional coupling between the left frontal and parietal cortex exhibited the strongest task-related modulation, with positive fronto-parietal coupling being significant across all n-back conditions. Moreover, brain-behavior correlations offered complementary insights. Specifically, the positive modulation of left IPL-to-IFG connectivity was negatively correlated with behavioral performance under the highest load condition, suggesting that individuals who performed better on the 3-back task required less positive parietal-to-frontal coupling for efficient performance. This may indicate that accurate and efficient VWM performance may require less input from the storage area in the inferior parietal cortex.^19^ However, the fact that this correlation was evident only in the 3-back condition suggests that IPL→IFG coupling only becomes behaviorally relevant under high working-memory load. Such a pattern may reflect load-dependent neural efficiency, whereby differences in network efficiency become behaviorally consequential only when demands are maximal, or compensatory or inefficient recruitment, in which poorer performers over-engage the IPL-IFG pathway to sustain performance. Alternatively, task-specific control engagement may be involved, as the 3-back condition uniquely taxes executive and interference-control processes supported by fronto-parietal communication. Another possibility is that ceiling and variance effects contribute, given that behavioral variability is greatest at high load.

Collectively, our DCM results reinforce the interpretation that the fronto-parietal axis plays a critical role in maintaining task-relevant information and managing cognitive resources during VWM processing.^44^ Moreover, effective connectivity was observed to be bi-directional, involving both anterior-to-posterior and posterior-to-anterior pathways, not only from parietal to frontal regions as shown in earlier studies.^53^ This bi-directionality underscores the dynamic and reciprocal nature of information flow during VWM tasks, wherein bottom-up sensory inputs and top-down goal- or attention-driven processes continuously interact to support encoding, maintenance, and retrieval.^54^^,^^55^^,^^56^

Last, DCM analyses revealed session-specific effects. In particular, a notable down-regulation of effective connectivity was observed when the task was performed for the first time. However, during the second session, this down-regulation was no longer evident, suggesting a form of neural adaptation or learning effect. The reversal to baseline connectivity in the second session may reflect increased efficiency or the establishment of more stable task-related network configurations following prior exposure. Interestingly, the driving input for all 1-, 2-, and 3-back conditions originated in the left IFG, underscoring its pivotal role in VWM. In this DCM, the driving input for the 2-back also entered the left IPL, providing evidence for its role in attentional control at intermediate working memory load, where demands are high but performance remains preserved.

### Limitations of the study

The current study has a few shortcomings we would like to address here. First, the sample size is inherently limited as it is derived from a multi-center study. Second, the effective connectivity analyses are based on a limited set of ROIs, derived primarily from prior literature. We acknowledge that the VWM network is not restricted to these six ROIs and involves other core nodes as well. While there is no general limitation as to how many regions one may include in a DCM, practical constraints such as computational cost, parameter-to-data ratio, and the Bayesian penalty for model complexity typically limit most studies to around 4–6 regions. Beyond that range, the risk of overfitting increases, model evidence tends to drop, and results become harder to interpret.^57^^,^^58^^,^^59^ Consequently, we restricted our model to the selected six ROIs. Since our ROI selection was motivated by those regions that showed strong increases in task-related activity, we are confident that this selection should provide a valid model of fronto-parieto-cerebellar interactions during VWM.

## Resource availability

### Lead contact

Requests for further information and resources should be directed to and will be fulfilled by the lead contact, Sabrina Turker (sabrina.turker@univie.ac.at).

### Materials availability

This study did not generate new unique reagents.

### Data and code availability

• **Data:** All raw and processed data generated in this study have been deposited at the OSF Platform and are publicly available at https://osf.io/x94c2/. Additional data supporting the findings of this study are available upon request from the corresponding author.
• **Code:** All custom code and scripts used for data processing and analysis have been deposited at GitHub at https://github.com/ggerardos/vwm-task-dcm-memoslap and are also publicly available through the OSF Platform at https://osf.io/x94c2/.
• **Other** **Items:** No supplementary software, algorithms, or related materials were developed as part of this study. All known and used software is cited in 10.3 and 10.4. Any additional information required to reanalyze the data reported in this paper is available from the corresponding author upon request.

## Acknowledgments

We would like to thank all participants and all student assistants who were involved in data collection and analysis. This research was funded by the 10.13039/501100001659German Research Foundation (Research Unit 5429/1 (467143400), HA 6314/10–1 to GH), the 10.13039/501100000781European Research Council (ERC consolidator grant FLEXBRAIN, ERC- COG-2021-101043747), and Lise Meitner Excellence funding by the 10.13039/501100004189Max Planck Society.

## Author contributions

Conceptualization: G.H.; data curation: S.T., G.G., and F.B.; formal analysis: G.G., F.B., and P.K.; funding acquisition: G.H.; investigation: S.T., G.G., and F.B.; methodology: G.H., S.T., G.G., F.B., and B.F.; project administration: S.T. and G.G.; resources: G.H.; supervision: G.H.; visualization: S.T. and G.G.; writing – original draft: S.T.; writing – review and editing: S.T., G.H., G.G., F.B., and P.K. All authors have read and approved the final version of this manuscript.

## Declaration of interests

The authors declare no competing interests.

## STAR★Methods

### Key resources table

**Table:** 

REAGENT or RESOURCE	SOURCE	IDENTIFIER
**Depositeddata**		

Raw and analyzed data	This paper	https://osf.io/x94c2/

**Softwareandalgorithms**		

PsychoPy	Peirce 2007^60^	https://doi.org/10.1016/j.jneumeth.2006.11.017
RStudio	R Core Team 2022	https://www.R-project.org/
Statistical Parametric Mapping (SPM12)	UCL	http://ww.fil.ion.ucl.ac.uk/spm/
fMRIprep (24.1.1)	Esteban et al. 2019^61^	https://fmriprep.org/en/stable/
BrainNet Viewer	Xia et al. 2013^62^	https://www.nitrc.org/projects/bnv/
MRIcroGL	Roden & Brett 2000^63^	(https://www.nitrc.org/projects/mricrogl)
FMRIB Software Library (FSL)	Jenkinson et al. 2012^64^	https://doi.org/10.1016/j.neuroimage.2011.09.015
Dynamic Causal Modeling (DCM)	Friston et al. 2003^65^	https://doi.org/10.1016/S1053-8119(03)00202-7
FLIRT	Diedrichsen et al. 2009^66^	https://doi.org/10.1016/j.neuroimage.2009.01.045

### Experimental model and study participants details

The present study reports findings of 20 healthy adults (M_age_ = 30.75 ± 7.5 years; age range: 18–45 years). 10 subjects were female (sex assigned at birth and gender identity). All participants were right-handed German native speakers with no previous history of neurological conditions or head injury. Details on race, ethnicity and ancestry are provided in Table S2: all participants reported being born and raised in Germany and identified as White/Middle European. Socio-economic status was not systematically assessed, but all participants completed at least 12 to 13 years of education, with only one subject possessing a PhD degree. Most participants were university students in their bachelor’s and master’s degrees. Participants were recruited using the subject database of the Max Planck Institute for Human Cognitive and Brain Sciences and through flyers that were distributed at Leipzig University. Participants were informed of the aim and purpose of the study and gave written informed consent before the experiment. The study was approved by the Ethics committee of the Medical Faculty at Leipzig University on March 28, 2022 (Study approval number: 085/22-ek).

This study describes the results of the preparatory phase of a larger, ongoing multicenter, double-blinded, sham-controlled crossover transcranial direct current stimulation study investigating the behavioral and neural effects of tDCS on learning and memory (https://www.memoslap.de/de/forschung/). Please note that all participants received placebo tDCS during the fMRI sessions since this cohort served as a control group. The sample size was determined within the larger multicenter study to determine session stability versus variability since most of the included paradigms used sample sizes ≤20 participants in previous studies. Please note that there were no sex-specific differences in VWM performance, both in terms of sensitivity and response times, which is why no further sex-specific analyses were performed.

### Method details

#### Experimental procedure

All participants were initially screened via phone or mail before participating in a behavioral testing session and two fMRI sessions. During the behavioral testing session all participants performed a 10-min practice session for the n-back task, which was identical to the final version of the task but only comprised half of the experimental blocks to familiarize them with the task. At least one week later, participants completed two fMRI task sessions, in which they performed the n-back task inside the scanner. The two task sessions were separated by at least seven days.

#### Task details

The n-back task was presented in a blocked design, randomly alternating between blocks of experimental conditions (1-back, 2-back and 3-back) and a control condition (0-back). Each of the 21 blocks (0-back: 3 blocks; 1-back: 6 blocks; 2-back: 6 blocks; 3-back: 6 blocks) contained 35 letters, with a randomized ratio of targets to non-targets between 1/3 and 1/4 (i.e., 9–11 matching targets per block and a total of 120 targets across blocks). Inter-block intervals were randomly jittered at 6.75, 11.25 or 16.25 s, with selection probabilities of 0.5, 0.3 and 0.2, respectively. This resulted in a total scanning time of ∼23 min. During all conditions, a series of white letters was presented on a mid-grey background with a text height of 0.15 (i.e., 15% of the display height). The letters were presented for 0.5 s in the middle of the screen followed by a blank screen (response time window: 1 s). In the 0-back condition, participants were instructed to press a button whenever the letter “X” was presented. In the three experimental conditions, participants had to press a button whenever a presented letter (target) was identical to a letter presented 1, 2 or 3 trials before. Participants had to respond to the relevant stimuli (targets) and withhold a response to non-targets (see Figure 1A). The task was presented using the software PsychoPy (v2023.1.2).^60^

#### Functional neuroimaging

fMRI data was collected on a 3T Prisma scanner (Siemens, Erlangen, Germany) with a 64-channel head coil. Blood oxygenation level-dependent (BOLD) images were acquired using a slice-accelerated multiband echo-planar imaging sequences (EPI) (TR = 1s, TE = 30.8 ms, flip angle = 60°, field of view = 220 mm, voxel size = 2 × 2 × 2mm, bandwidth = 2165/Px, phase encoding direction = anterior-posterior (AP)).

### Quantification and statistical analysis

#### Behavioral, statistical analyses

All behavioral analyses were performed in R using RStudio.^67^ For the n-back task, three behavioral outcome measures were computed, namely accuracy, response times and sensitivity (*d’*). The *d’* prime score was calculated from hits and false alarms: *d’* = z(hits) – z(false alarms). This score indicates whether participants pressed the button when they were supposed to press (target) and did not press when they were not supposed to press (non-target). By considering both performance for targets and non-targets (i.e., the ability to detect targets but ignore irrelevant information), it is considered a more fine-grained measure than pure accuracy.^68^ Additionally, hit and false-alarm rates were adjusted^69^ to correct for perfect or zero performance values that can otherwise produce infinite estimates and distort sensitivity measures.

To compare overall mean performance (*d′/*response times - RT) between the n-back conditions across all sessions, we applied the Friedman rank-sum test, followed by post-hoc Wilcoxon signed-rank tests with Bonferroni correction. The behavioral results for sensitivity and response times are shown in Figures 1B and 1C. To examine differences in mean performance (d′/RT) between sessions, we used a 2 × 4 ART ANOVA (aligned rank transform; non-parametric version of multi-level ANOVA), with the factors condition (1-back, 2-back, 3-back) and session (session 1, session 2) and their interaction, followed by post-hoc Wilcoxon signed-rank tests with Bonferroni correction. The stability of the behavioral results in the two consecutive sessions is shown in Figures 1D and 1E.

#### fMRI analysis

fMRI data were analyzed using Statistical Parametric Mapping (SPM12; Wellcome Trust Center for Neuroimaging; http://ww.fil.ion.ucl.ac.uk/spm/) implemented in MATLAB (version 24.1.0/2024a). Data were organized using the BIDS (Brain Data Imaging Structure; https://bids.neuroimaging.io/index.html) format^69^ and preprocessed using fMRIprep (24.1.1).^61^ Functional images were realigned, corrected for distortion, slice-time corrected and normalized to MNI space. Then, they were smoothed with a 4 mm FWHM Gaussian kernel. All functional images were masked with a whole-brain inclusive mask generated by fMRIprep.

Whole-brain functional activation was analyzed via random-effects group analyses based on the GLM, using the classical two-level approach in SPM12. For this purpose, both task sessions were combined with diagonal concatenation for further analysis. The GLM included regressors for the four experimental conditions (0-back, 1-back, 2-back, 3-back), and instructions were modeled as a separate regressor. Trials were specified in a blocked design and convolved with the canonical hemodynamic response function (HRF). In addition, nuisance regressors were included in the subject-level GLM to control for non-neural sources of variance: 24 motion parameters (i.e., the 6 motion parameters, as well as their squares, derivatives, and squares of derivatives), 10 aCompCor principal components (from white matter and cerebrospinal fluid), a binary regressor indicating volumes with framewise displacement ≥0.9, a mean-centered block identifier, and a binary regressor marking blocks with d′ ≤ 0. First-level contrast images were computed for each subject. These contrasts were then entered into one-sample t-tests at the second (group) level. All n-back levels were contrasted with the baseline (0-back). Additionally, two contrasts averaging activity across all VWM conditions relative to the baseline (1 + 2+3-back > 0-back and 0-back >1 + 2+3-back) were computed. All activation maps were corrected at a voxel-wise *p* < 0.001 and a cluster-wise *p* < 0.05 FWE-corrected. Figures in the present manuscript were created using BrainNet Viewer^62^ and MRIcroGL.^63^ We repeated this analysis modeling the 1-back, 2-back and 3-back conditions as a single regressor, with the VWM load (1–3) entered as mean centered parametric modulator. We then inspected the parametric effects (positive and negative) against the intrinsic baseline.

To quantify the similarity between the task-related activation brain map with the MDN and DMN we used the overlap coefficient (or Szymkiewicz–Simpson coefficient). This metric is particularly appropriate for comparing maps of unequal size as it captures the containment of the smaller map within the bigger one. It is defined as the number of shared elements (i.e., voxels) divided by the total number of elements of the smaller map. The overlap coefficient ranges from 0 to 1 (no overlap) and 1 (complete containment of one of the maps into the other.^70^ To create the activation maps, we binarized the T-maps of the 1 + 2+3-back > 0-back and 0 > 1+2+3-back contrasts and thresholding voxels at *p* < 0.001 uncorrected in combination with cluster level (FWE) correction at *p* < 0.05. Finally, we co-registered and resliced binary versions of the network masks (MDN and DMN) to the activation maps.^34^^,^^35^

#### ROI selection

We selected functional regions of interest (fROIs) for the effective connectivity analysis based on theoretical assumptions and existing empirical evidence demonstrating their specific involvement in the VWM network. The bilateral IFG was included given its role in the articulatory loop and its importance for articulatory rehearsal and updating processes.^15^^,^^71^ The bilateral IPL was chosen because of its function as a phonological storage buffer and convergence zone supporting both domain-specific and domain-general processes.^14^^,^^20^ Finally, the bilateral superior cerebellum (crus I, lobule VI) was selected based on its reciprocal connectivity with frontal and parietal regions and its sensitivity to load differences during VWM tasks.^15^^,^^24^^,^^72^

Activation magnitudes for each selected fROI were calculated for each participant as the mean value of the top 10% of voxels with the highest t-values in the task-general contrast (1 + 2+3-back > 0-back). Comparisons of activation magnitudes across n-back conditions were performed using the Friedman rank-sum test, with post-hoc Dunn–Šidák comparisons.

#### Effective connectivity analysis

Based on the activation results, we performed a group-constrained, subject-specific functional ROI (fROI) analysis. We chose the 1 + 2+3-back > 0-back as effect-of-interest. Next, the activation maps for this contrast were thresholded individually for each subject at a *p*-value of *p* < 0.0001. Then, these individual activation maps were overlayed to create a probabilistic overlap map, which was then smoothed with a 6 mm FWHM Gaussian kernel. We set the minimum number of participants to show an effect in each voxel to two participants (10% of the overall sample). To restrict the fROI search to anatomical regions of interest, we used a binary mask created using the Harvard-Oxford Atlas^73^ for cortical regions and the Cerebellar Atlas in MNI152 space after normalization with FLIRT.^66^

To extract the relevant regions (bilateral IFG, bilateral IPL and bilateral Cerebellum), the FMRIB Software Library (FSL)^64^^,^^74^ was used. Next, the extracted cortical regions were thresholded (>30% probability) and summarized in a single ROI image. Similarly, the extracted cerebellar image was thresholded (>1% probability) and binarized. Then both binary masks were put together and the mask was co-registered (resliced) with SPM12 to fit the overlap map (Figure S1A). This overlap map was then parcellated with a watershed algorithm^75^ and the resulting parcellation was thresholded to only retain regions where at least 80% of participants showed significantly activated voxels. This parcellation resulted in multiple sub-regions, from which only one was selected per pre-defined relevant region. In the cortex, sub-regions with a 100% subject overlap and larger volume (more voxels) were preferred. In the cerebellum, the same criteria were used, but the selection was restricted to the superior portion of the cerebellum to focus on those areas shown to be sensitive to load differences during VWM tasks in our fMRI analysis (Figure S1B). For each subject, individual ROIs were defined by selecting the top 10% of the voxels within each general ROI that showed the highest positive t-value for the 123-back > 0-back contrast (Figure S1C).

To explore directed coupling between the selected ROIs, we used Dynamic Causal Modeling (DCM).^65^ DCM analyses were performed with SPM12 and MATLAB (version 24.1.0/2024a). First, we concatenated the two sessions for the GLM analysis to obtain a single continuous time series using spm_fmri_concatenate(), which implements the appropriate adjustments for multi-session analyses; it models session-specific high-pass filtering, temporal autocorrelation, and includes run-specific regressors to account for differences in signal and potential drifts between sessions. An F contrast (identity matrix across the n-back regressors) was calculated to capture the variance explained by any of the conditions; this was used as a general effects measure. We then performed a Volume of Interest (VOI) analysis to extract the first eigenvariate of the BOLD time series from each individual ROI (bilateral IFG, bilateral IPL, and bilateral cerebellum; adjusted for effects of interest). These time series were the input of our DCM model. The final step, before calculating the DCM model, was to construct a new DCM-specific GLM to specify the experimental inputs to the model. This GLM included separate regressors for all task conditions (0-back, 1-back, 2-back, and 3-back) and one regressor that combined the onsets and timings of all blocks (all-blocks condition or “all conditions”).

The DCM analysis was performed using the Parametric Empirical Bayes (PEB) framework.^58^^,^^76^ At the first level, a full model was constructed and estimated for each subject. The full model contained all six ROIs with reciprocal connections. The onsets of the all-blocks condition were set as the driving input of all ROIs. The 1-, 2-, and 3-back conditions were specified as modulatory inputs expected to influence the connectivity between the ROIs. The DCM inputs were mean-centered such that the intrinsic connectivity represented the mean connectivity across all experimental conditions (task general connectivity), while the modulation parameters captured condition-specific deviations from this task general connectivity.^57^ A maximum of 512 iterations was allowed to ensure that all subject models converged to a stable optimum solution. At the second level, individual differences in connection strengths were decomposed into group effects and random effects using PEB. The resulting Reduced Connectivity Models (RCMs) provided subject-specific parameter estimates informed by the group model. Bayesian Model Reduction (BMR) was then employed to compare the full model against 256 reduced models to cut parameters that do not contribute to the model evidence. Finally, Bayesian Model Averaging (BMA) was performed, and parameters were thresholded to retain only those with a posterior probability of Pp > 0.99 The BMA subsequently allowed inspection and interpretation of parameters of the model that best explained the data. To test for potential session effects in effective connectivity, we repeated this analysis with a different DCM-specific GLM; we included separate regressors for all task conditions (0-back, 1-back, 2-back, and 3-back) but one regressor per session that combined the onsets and timings of all blocks of that session (session-1 and session-2). The session-1 and session-2 conditions were modeled as modulatory inputs, while the onsets of the 1-, 2-, and 3-back conditions were set as separate driving inputs. The rest of the analysis remained the same.

To calculate and inspect the changes in effective connectivity per condition after modulation (in Hz), we used formula 3 in Zeidman et al.^57^ To explore the relationship between effective connectivity and task performance, we extracted subject-specific estimates of all intrinsic connections and modulatory effects from the RCMs; only parameters that survived BMA with a posterior probability Pp > 0.99 were tested. Intrinsic connections were correlated with mean sensitivity (d′) and reaction time of correct trials, averaged across all conditions and weighted by the ratio of blocks per condition. Modulatory strengths were correlated with mean sensitivity (d′) and reaction time of correct trials in the corresponding condition. We used Bayesian correlation because it provides more stable estimates in smaller samples, as it does not rely on large-sample approximations but instead combines prior information with the observed data to yield full posterior distributions of the correlation coefficients. Evidence for or against associations is then directly quantified via Bayes factors, which represent the relative evidence for one model (e.g., the presence of a correlation) compared to another (e.g., no correlation). As we had no prior hypotheses regarding the direction of the relationships, we adopted the more conservative two-sided test BF_10_. Interpretation of BF_10_ values followed the guidelines by Lee and Wagenmakers.^77^

## References

[1] Abellaneda-Pérez K., Vaqué-Alcázar L., Perellón-Alfonso R., Bargalló N., Kuo M.-F., Pascual-Leone A., Nitsche M.A., Bartrés-Faz D. (2020). Differential tDCS and tACS Effects on Working Memory-Related Neural Activity and Resting-State Connectivity. Front. Neurosci..

[2] Johnson M.K., McMahon R.P., Robinson B.M., Harvey A.N., Hahn B., Leonard C.J., Luck S.J., Gold J.M. (2013). The relationship between working memory capacity and broad measures of cognitive ability in healthy adults and people with schizophrenia. Neuropsychology.

[3] Wiley J., Jarosz A.F. (2012). Working Memory Capacity, Attentional Focus, and Problem Solving. Curr. Dir. Psychol. Sci..

[4] Craik F.I.M., Eftekhari E., Bialystok E., Anderson N.D. (2018). Individual differences in executive functions and retrieval efficacy in older adults. Psychol. Aging.

[5] Park D.C., Reuter-Lorenz P. (2009). The Adaptive Brain: Aging and Neurocognitive Scaffolding. Annu. Rev. Psychol..

[6] Pliatsikas C., Veríssimo J., Babcock L., Pullman M.Y., Glei D.A., Weinstein M., Goldman N., Ullman M.T. (2019). Working memory in older adults declines with age, but is modulated by sex and education. Q. J. Exp. Psychol..

[7] Lee J., Park S. (2005). Working Memory Impairments in Schizophrenia: A Meta-Analysis. J. Abnorm. Psychol..

[8] Nakao T., Nakagawa A., Nakatani E., Nabeyama M., Sanematsu H., Yoshiura T., Togao O., Tomita M., Masuda Y., Yoshioka K. (2009). Working memory dysfunction in obsessive–compulsive disorder: A neuropsychological and functional MRI study. J. Psychiatr. Res..

[9] Baddeley A. (1992). Working Memory. Science.

[10] Buchsbaum B.R., Hickok G., Small S.L. (2016). Neurobiology of Language.

[11] Archibald L.M.D. (2013). The Language, Working Memory, and Other Cognitive Demands of Verbal Tasks. Top. Lang. Disord..

[12] Hughes R.W. (2025). The phonological store of working memory: A critique and an alternative, perceptual-motor, approach to verbal short-term memory. Q. J. Exp. Psychol..

[13] Towse J., Hurlstone M., Atkinson A., Saito S., Logie R. (2025). Working memory gets a workout: Reviewing the legacy of Baddeley and Hitch (1974) 50 years on. Q. J. Exp. Psychol..

[14] Buchsbaum B.R., D’Esposito M. (2019). A sensorimotor view of verbal working memory. Cortex.

[15] Emch M., von Bastian C.C., Koch K. (2019). Neural Correlates of Verbal Working Memory: An fMRI Meta-Analysis. Front. Hum. Neurosci..

[16] Owen A.M., McMillan K.M., Laird A.R., Bullmore E. (2005). N-back working memory paradigm: A meta-analysis of normative functional neuroimaging studies. Hum. Brain Mapp..

[17] Rottschy C., Langner R., Dogan I., Reetz K., Laird A.R., Schulz J.B., Fox P.T., Eickhoff S.B. (2012). Modelling neural correlates of working memory: A coordinate-based meta-analysis. Neuroimage.

[18] Wager T.D., Smith E.E. (2003). Neuroimaging studies of working memory. Cogn. Affect. Behav. Neurosci..

[19] Jonides J., Schumacher E.H., Smith E.E., Koeppe R.A., Awh E., Reuter-Lorenz P.A., Marshuetz C., Willis C.R. (1998). The Role of Parietal Cortex in Verbal Working Memory. J. Neurosci..

[20] Paulesu E., Frith C.D., Frackowiak R.S. (1993). The neural correlates of the verbal component of working memory. Nature.

[21] Smith E.E., Jonides J. (1997). Working Memory: A View from Neuroimaging. Cogn. Psychol..

[22] Fiez J.A., Hickok G., Small S.L. (2016). Neurobiology of Language.

[23] Ashida R., Cerminara N.L., Edwards R.J., Apps R., Brooks J.C.W. (2019). Sensorimotor, language, and working memory representation within the human cerebellum. Hum. Brain Mapp..

[24] Peterburs J., Liang Y., Cheng D.T., Desmond J.E. (2021). Sensory acquisition functions of the cerebellum in verbal working memory. Brain Struct. Funct..

[25] Sheu Y.S., Liang Y., Desmond J.E. (2019). Disruption of Cerebellar Prediction in Verbal Working Memory. Front. Hum. Neurosci..

[26] Lesage E., Hansen P.C., Miall R.C. (2017). Right Lateral Cerebellum Represents Linguistic Predictability. J. Neurosci..

[27] Argyropoulos G.P.D. (2016). The cerebellum, internal models and prediction in ‘non-motor’ aspects of language: A critical review. Brain Lang..

[28] Chen S.H.A., Desmond J.E. (2005). Temporal dynamics of cerebro-cerebellar network recruitment during a cognitive task. Neuropsychologia.

[29] Marvel C.L., Desmond J.E. (2010). The contributions of cerebro-cerebellar circuitry to executive verbal working memory. Cortex.

[30] Turker S., Fumagalli B., Kuhnke P., Hartwigsen G. (2025). The ‘reading’ brain: Meta-analytic insight into functional activation during reading in adults. Neurosci. Biobehav. Rev..

[31] Turker S., Kuhnke P., Eickhoff S.B., Caspers S., Hartwigsen G. (2023). Cortical, subcortical, and cerebellar contributions to language processing: A meta-analytic review of 403 neuroimaging experiments. Psychol. Bull..

[32] Kirschen M.P., Chen S.H.A., Schraedley-Desmond P., Desmond J.E. (2005). Load- and practice-dependent increases in cerebro-cerebellar activation in verbal working memory: an fMRI study. Neuroimage.

[33] Hampson M., Driesen N.R., Skudlarski P., Gore J.C., Constable R.T. (2006). Brain Connectivity Related to Working Memory Performance. J. Neurosci..

[34] Darda K.M., Butler E.E., Ramsey R. (2018). Functional Specificity and Sex Differences in the Neural Circuits Supporting the Inhibition of Automatic Imitation. J. Cogn. Neurosci..

[35] Schaefer A., Kong R., Gordon E.M., Laumann T.O., Zuo X.N., Holmes A.J., Eickhoff S.B., Yeo B.T.T. (2018). Local-global parcellation of the human cerebral cortex from intrinsic functional connectivity mri. Cereb. Cortex.

[36] Wen T., Duncan J., Mitchell D.J. (2020). Hierarchical Representation of Multistep Tasks in Multiple-Demand and Default Mode Networks. J. Neurosci..

[37] Raichle M.E. (2015). The Brain’s Default Mode Network. Annu. Rev. Neurosci..

[38] Smallwood J., Bernhardt B.C., Leech R., Bzdok D., Jefferies E., Margulies D.S. (2021). The default mode network in cognition: a topographical perspective. Nat. Rev. Neurosci..

[39] Barrouillet P., Bernardin S., Camos V. (2004). Time Constraints and Resource Sharing in Adults’ Working Memory Spans. J. Exp. Psychol. Gen..

[40] Barrouillet P., Bernardin S., Portrat S., Vergauwe E., Camos V. (2007). Time and cognitive load in working memory. J. Exp. Psychol. Learn. Mem. Cogn..

[41] Langerock N., Oberauer K., Throm E., Vergauwe E. (2025). The cognitive load effect in working memory: Refreshing the empirical landscape, removing outdated explanations. J. Mem. Lang..

[42] Camilleri J.A., Müller V.I., Fox P., Laird A.R., Hoffstaedter F., Kalenscher T., Eickhoff S.B. (2018). Definition and characterization of an extended Multiple-Demand Network. Neuroimage.

[43] Menon V. (2023). 20 years of the default mode network: A review and synthesis. Neuron.

[44] Assem M., Blank I.A., Mineroff Z., Ademoğlu A., Fedorenko E. (2020). Activity in the fronto-parietal multiple-demand network is robustly associated with individual differences in working memory and fluid intelligence. Cortex.

[45] Leung A.W.S., Moreno S., Alain C. (2023). The frontoparietal multiple demand network interacts with the dual pathways in auditory working memory. Cereb. Cortex.

[46] Shashidhara S., Mitchell D.J., Erez Y., Duncan J. (2019). Progressive Recruitment of the Frontoparietal Multiple-demand System with Increased Task Complexity, Time Pressure, and Reward. J. Cogn. Neurosci..

[47] Wen T., Egner T. (2023). Context-independent scaling of neural responses to task difficulty in the multiple-demand network. Cereb. Cortex.

[48] Koshino H., Minamoto T., Yaoi K., Osaka M., Osaka N. (2014). Coactivation of the Default Mode Network regions and Working Memory Network regions during task preparation. Sci. Rep..

[49] Duncan J. (2010). The multiple-demand (MD) system of the primate brain: mental programs for intelligent behaviour. Trends Cogn. Sci..

[50] Chrabaszcz A., Lear K., Durisko C., Fiez J.A. (2025). Contributions of the multiple demand network to emergent and skilled reading. Sci. Rep..

[51] Saadon-Grosman N., Du J., Kosakowski H.L., Angeli P.A., DiNicola L.M., Eldaief M.C., Buckner R.L. (2024). Within-individual organization of the human cognitive cerebellum: Evidence for closely juxtaposed, functionally specialized regions. Sci. Adv..

[52] D’Mello A.M., Gabrieli J.D.E., Nee D.E. (2020). Evidence for Hierarchical Cognitive Control in the Human Cerebellum. Curr. Biol..

[53] Ma L., Steinberg J.L., Hasan K.M., Narayana P.A., Kramer L.A., Moeller F.G. (2012). Working memory load modulation of parieto-frontal connections: Evidence from dynamic causal modeling. Hum. Brain Mapp..

[54] Edin F., Klingberg T., Johansson P., McNab F., Tegnér J., Compte A. (2009). Mechanism for top-down control of working memory capacity. Proc. Natl. Acad. Sci..

[55] Zanto T.P., Rubens M.T., Thangavel A., Gazzaley A. (2011). Causal role of the prefrontal cortex in top-down modulation of visual processing and working memory. Nat. Neurosci..

[56] Zheng S., Zhang Y., Huang K., Zhuang J., Lü J., Liu Y. (2025). Temporal Interference Stimulation Boosts Working Memory Performance in the Frontoparietal Network. Hum. Brain Mapp..

[57] Zeidman P., Jafarian A., Corbin N., Seghier M.L., Razi A., Price C.J., Friston K.J. (2019). A guide to group effective connectivity analysis, part 1: First level analysis with DCM for fMRI. Neuroimage.

[58] Friston K.J., Preller K.H., Mathys C., Cagnan H., Heinzle J., Razi A., Zeidman P. (2019). Dynamic causal modelling revisited. Neuroimage.

[59] Stephan K.E., Penny W.D., Moran R.J., den Ouden H.E.M., Daunizeau J., Friston K.J. (2010). Ten simple rules for dynamic causal modeling. Neuroimage.

[60] Peirce J.W. (2007). PsychoPy—Psychophysics software in Python. J. Neurosci. Methods.

[61] Esteban O., Markiewicz C.J., Blair R.W., Moodie C.A., Isik A.I., Erramuzpe A., Kent J.D., Goncalves M., DuPre E., Snyder M. (2019). fMRIPrep: a robust preprocessing pipeline for functional MRI. Nat. Methods.

[62] Gorgolewski K.J., Auer T., Calhoun V.D., Craddock R.C., Das S., Duff E.P., Flandin G., Ghosh S.S., Glatard T., Halchenko Y.O. (2016). The brain imaging data structure, a format for organizing and describing outputs of neuroimaging experiments. Sci. Data.

[63] Rorden C., Brett M. (2000). Stereotaxic display of brain lesions. Behav. Neurol..

[64] Jenkinson M., Beckmann C.F., Behrens T.E.J., Woolrich M.W., Smith S.M. (2012). FSL. Neuroimage.

[65] Friston K.J., Harrison L., Penny W. (2003). Dynamic causal modelling. Neuroimage.

[66] Diedrichsen J., Balsters J.H., Flavell J., Cussans E., Ramnani N. (2009). A probabilistic MR atlas of the human cerebellum. Neuroimage.

[67] R Core Team (2022). R: A language and environment for statistical computing. https://www.R-project.org/.

[68] Lynn S.K., Barrett L.F. (2014). “Utilizing” Signal Detection Theory. Psychol. Sci..

[69] Hautus M.J. (1995). Corrections for extreme proportions and their biasing effects on estimated values ofd. Behav. Res. Methods Instrum. Comput..

[70] Fuentes-Claramonte P., Santo-Angles A., Argila-Plaza I., Lechón M., Guardiola-Ripoll M., Almodóvar-Payá C., Cullen B., Evans J.J., Manly T., Gee A. (2021). Brain imaging of executive function with the computerised multiple elements test. Brain Imaging Behav..

[71] Fiez J.A., Raife E.A., Balota D.A., Schwarz J.P., Raichle M.E., Petersen S.E. (1996). A positron emission tomography study of the short-term maintenance of verbal information. J. Neurosci..

[72] Desmond J.E., Gabrieli J.D., Wagner A.D., Ginier B.L., Glover G.H. (1997). Lobular Patterns of Cerebellar Activation in Verbal Working-Memory and Finger-Tapping Tasks as Revealed by Functional MRI. J. Neurosci..

[73] Desikan R.S., Ségonne F., Fischl B., Quinn B.T., Dickerson B.C., Blacker D., Buckner R.L., Dale A.M., Maguire R.P., Hyman B.T. (2006). An automated labeling system for subdividing the human cerebral cortex on MRI scans into gyral based regions of interest. Neuroimage.

[74] Smith S.M., Jenkinson M., Woolrich M.W., Beckmann C.F., Behrens T.E.J., Johansen-Berg H., Bannister P.R., De Luca M., Drobnjak I., Flitney D.E. (2004). Advances in functional and structural MR image analysis and implementation as FSL. Neuroimage.

[75] Meyer F. (1994). Topographic distance and watershed lines. Signal Process..

[76] Friston K.J., Litvak V., Oswal A., Razi A., Stephan K.E., van Wijk B.C.M., Ziegler G., Zeidman P. (2016). Bayesian model reduction and empirical Bayes for group (DCM) studies. Neuroimage.

[77] Lee M.D., Wagenmakers E.J. (2014).

