# Supplementary material for: Task load modulates network interactions between bilateral fronto-parietal and cerebellar areas during verbal working memory
Source: iScience. 2026 Mar 26;29(5):115502. doi: 10.1016/j.isci.2026.115502 (PMC13091522; doi:10.1016/j.isci.2026.115502)
Supplement: Document S1. Figures S1–S3 and Tables S1 and S2 [file mmc1.pdf]

iScience, Volume *29*

Supplemental information

Task load modulates network interactions between bilateral fronto-parietal and cerebellar areas during verbal working memory

Sabrina Turker, Gerasimos Gerardos, Felix Büch, Beatrice Fumagalli, Philipp Kuhnke, and Gesa Hartwigsen

Supplementary Information

Figure S1. Specific details on the chosen fROIs, related to effective connectivity analysis (STAR methods section). (A) Anatomical masks of the ROIs selected for this study (bilateral IFG, bilateral IPL and bilateral cerebellum) based on the Harvard-Oxford Atlas [S1,S2] for cortical regions and the Cerebellar Atlas [S3]. **(B)** Selected sub-regions (fROIs) within the anatomical ROIs shown in A, defined via the watershed algorithm [S4]. **(C)** Constrained versions of the fROIs shown in B, including only the voxels within the top 10% of t-values in the 1+2+3-back contrast for at least 1 participant.

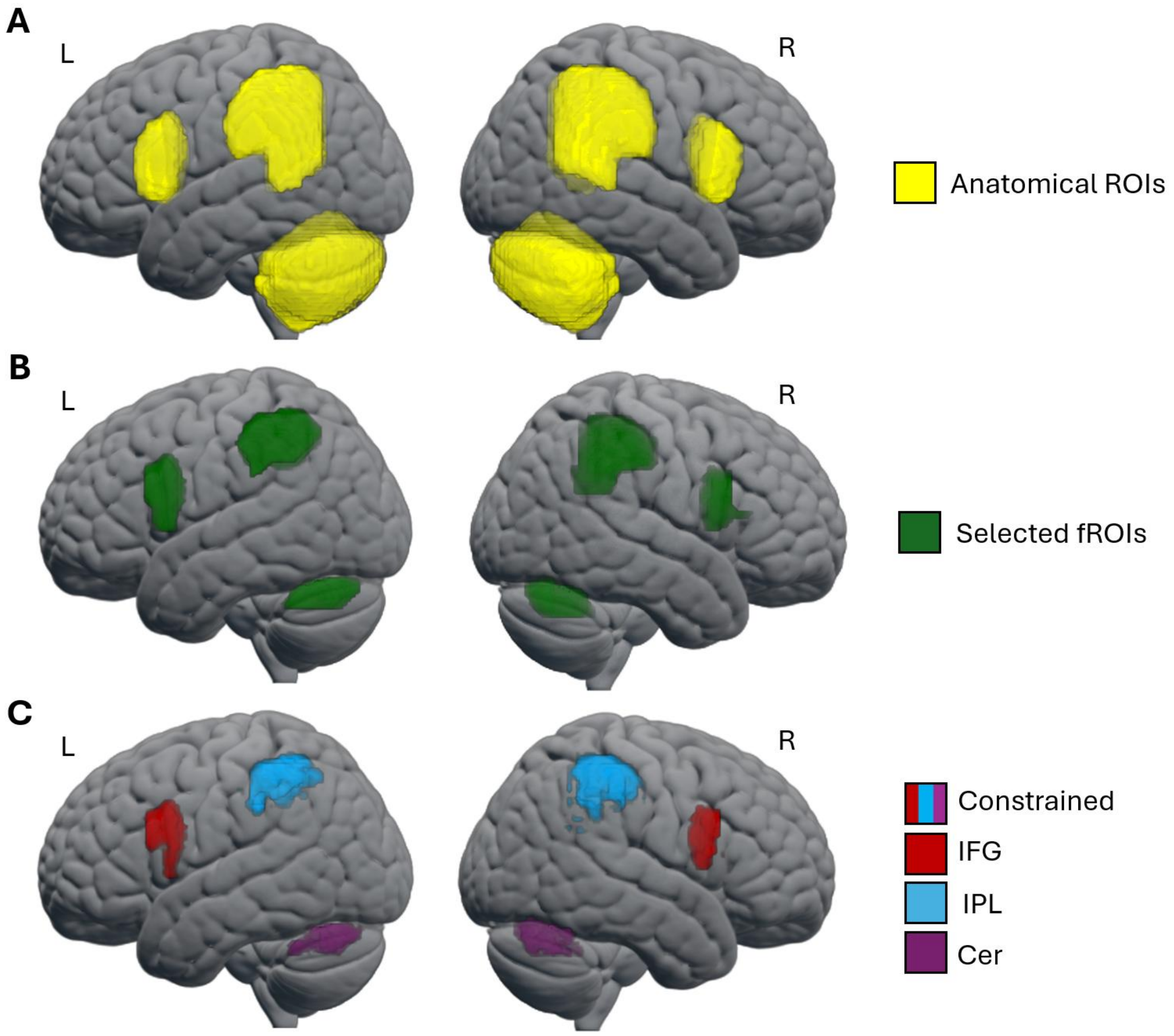

Figure S2. Parametric modulation of task load (1-, 2-, 3-back), related to Figure 3. Positive and negative effects of the parametric modulators against the intrinsic baseline are depicted. Results are shown at p<.001 uncorrected at the voxel level and p<.05 corrected at the cluster level (FWE).

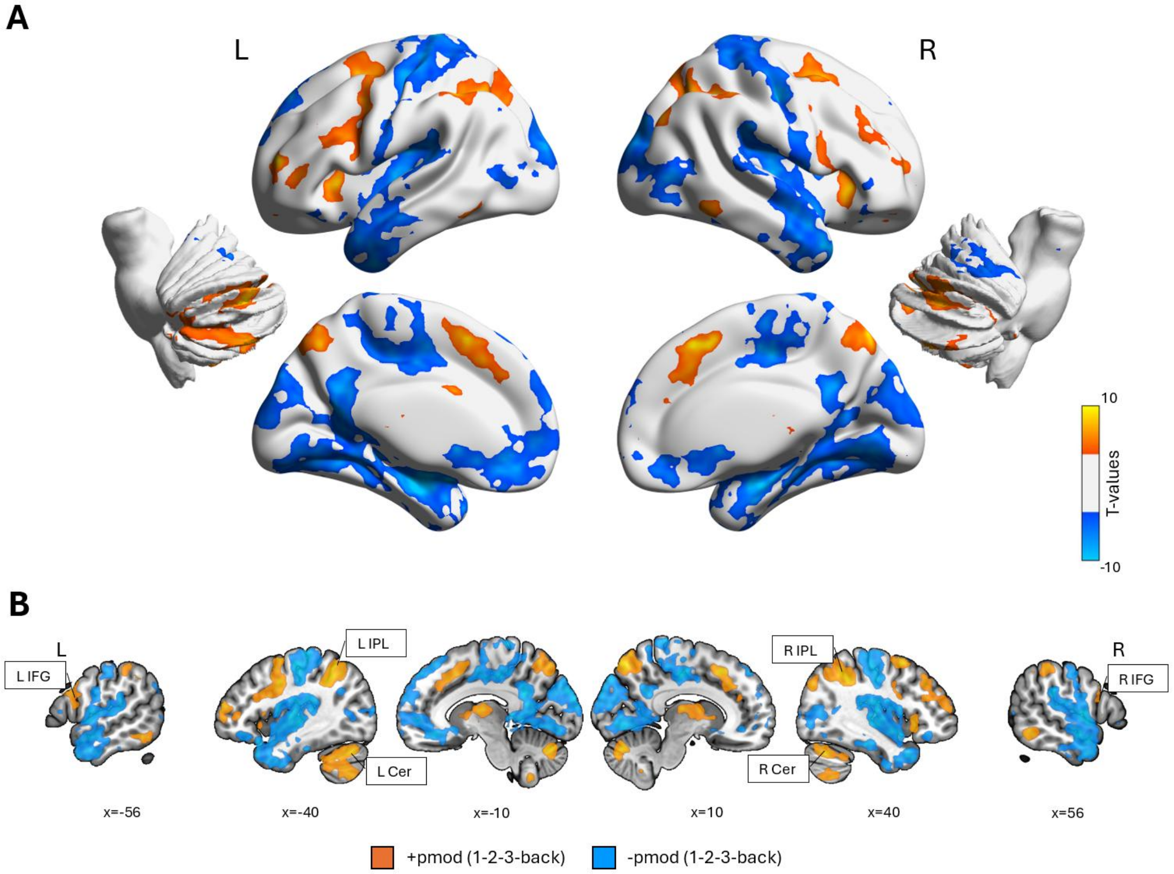

Figure S3. Full DCM models for effective connectivity analysis, related toTable 2 and Figure 4. (A) Full DCM model for the main analysis: specifying bi-directional intrinsic connections and task modulations (1-back, 2-back, 3-back) between six selected nodes of the VWM network: L IFG, R IFG, L IPL, R IPL, L CER and R CER. All trials served as driving input. **(B)** Full DCM to explore session-effects, specifying bi-directional intrinsic connections between the same regions and session (1 and 2) as modulatory effects. n-back levels were included as driving input. Self-connections were included for all regions in both analyses.

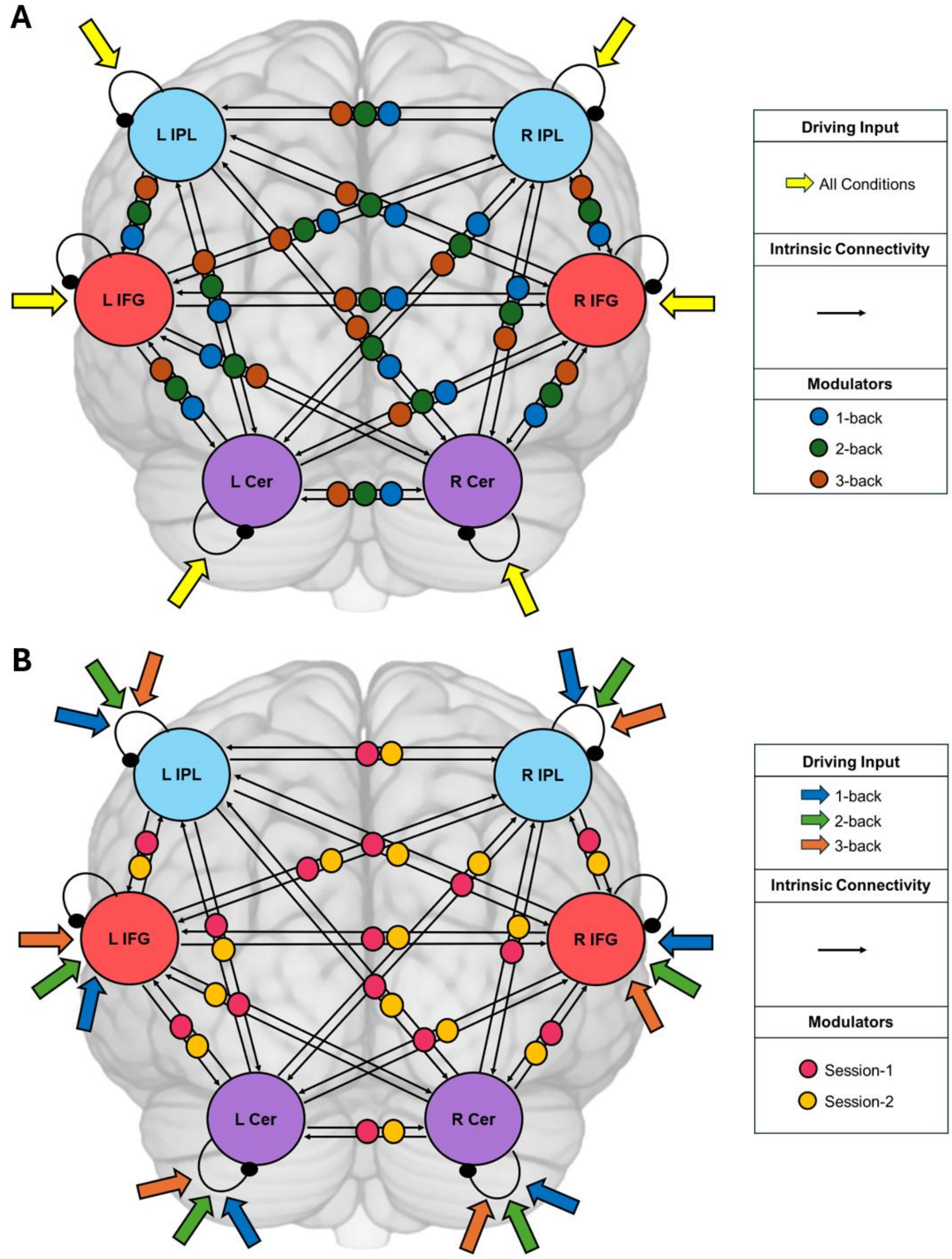

Table S1. Details of the behavioral results, related to Figure 1B-E. Repeated-measures ANOVA results for differences in sensitivity (d' prime) and response times for the different n-back conditions and between sessions, as well as the interaction between session and condition.

**Sensitivity**			
	F	***df***	***p***
condition	**221.64**	**3**	**<.001*****
session	**1.79**	**1**	**0.183**
condition x session	**0.61**	**3**	**0.607**
**Reaction times**			
	F	***df***	***p***
condition	**180**	**3**	**<.001*****
session	**1**	**1**	**0.32**
condition x session	**0.53**	**3**	**0.663**

Table S2. Demographic information of study participants. Details on age, sex/gender, ancestry, race and ethnicity are included for each study participant.

**Study-ID**	**age**	**sex/ gender**	**ancestry**	**race**	**ethnicity**
sub-4001p4	38	f	Middle European	White / Caucasian	German
sub-4002p4	29	f	Middle European	White / Caucasian	German
sub-4003p4	38	f	Middle European	White / Caucasian	German
sub-4004p4	24	m	Middle European	White / Caucasian	German
sub-4005p4	36	f	Middle European	White / Caucasian	German
sub-4006p4	40	m	Middle European	White / Caucasian	German
sub-4007p4	27	m	Middle European	White / Caucasian	German
sub-4008p4	34	m	Middle European	White / Caucasian	German
sub-4009p4	24	m	Middle European	White / Caucasian	German
sub-4010p4	27	f	Middle European	White / Caucasian	German

sub-4011p4	36	m	Middle European	White / Caucasian	German
sub-4012p4	20	f	Middle European	White / Caucasian	German
sub-4013p4	32	f	Middle European	White / Caucasian	German
sub-4014p4	36	m	Middle European	White / Caucasian	German
sub-4015p4	40	f	Middle European	White / Caucasian	German
sub-4016p4	44	m	Middle European	White / Caucasian	German
sub-4017p4	21	f	Middle European	White / Caucasian	German
sub-4018p4	23	m	Middle European	White / Caucasian	German
sub-4019p4	25	m	Middle European	White / Caucasian	German
sub-4020p4	21	f	Middle European	White / Caucasian	German
sub-4021p4	24	m	Middle European	White / Caucasian	German

Supplemental References

S1. Desikan, R.S., Ségonne, F., Fischl, B., Quinn, B.T., Dickerson, B.C., Blacker, D., Buckner, R.L., Dale, A.M., Maguire, R.P., Hyman, B.T., et al. (2006). An automated labeling system for subdividing the human cerebral cortex on MRI scans into gyral based regions of interest. NeuroImage *31*, 968–980. https://doi.org/10.1016/j.neuroimage.2006.01.021.

S2. Makris, N., Goldstein, J.M., Kennedy, D., Hodge, S.M., Caviness, V.S., Faraone, S.V., Tsuang, M.T., and Seidman, L.J. (2006). Decreased volume of left and total anterior insular lobule in schizophrenia. Schizophr. Res. *83*, 155–171. https://doi.org/10.1016/j.schres.2005.11.020.

S3. Diedrichsen, J., Balsters, J.H., Flavell, J., Cussans, E., and Ramnani, N. (2009). A probabilistic MR atlas of the human cerebellum. NeuroImage *46*, 39–46. https://doi.org/10.1016/j.neuroimage.2009.01.045.

S4. Meyer, F. (1994). Topographic distance and watershed lines. Signal Process. *38*, 113–125. https://doi.org/10.1016/0165-1684(94)90060-4.
